# *Arabidopsis* Transcription Factor MYB102 Increases Plant Susceptibility to Aphids by Substantial Activation of Ethylene Biosynthesis

**DOI:** 10.3390/biom8020039

**Published:** 2018-06-07

**Authors:** Lin Zhu, Jiansheng Guo, Zhongyou Ma, Jianfei Wang, Cheng Zhou

**Affiliations:** 1 College of Resource and Environment, Anhui Science and Technology University, Bengbu 233100, China; zhul@ahstu.edu.cn (L.Z.); mazy@ahstu.edu.cn (Z.M.); wangjf007@ahstu.edu.cn (J.W.); 2 School of Medicine, Zhejiang University, Hangzhou 310058, China; jsguo518@zju.edu.cn

**Keywords:** ethylene, *Myzus persicae*, aphid susceptibility, 1-aminocyclopropane-1-carboxylate synthase, host resistance

## Abstract

Induction of ethylene biosynthesis by aphids increases the susceptibility of several plant species to aphids. Recent studies have indicated that some MYB transcription factors regulate the phloem-based defense against aphid infestation by modulating ethylene (ET) signaling. *Arabidopsis MYB102* has previously been shown to be induced by wound signaling and regulate defense response against chewing insects. However, it remains unclear whether *Arabidopsis* *MYB102* takes part in the defense response of plants to aphids. Here, we investigated the function of *MYB102* in the response of *Arabidopsis* to aphid infestation. *Arabidopsis* *MYB102* was primarily expressed in vascular tissues, and its transcription was remarkably induced by green peach aphids (GPA; *Myzus persicae*). The results of RNA-Sequencing revealed that overexpression of *MYB102* in *Arabidopsis* promoted ET biosynthesis by upregulation of some 1-aminocyclopropane-1-carboxylate synthase (ACS) genes, which are rate-limiting enzymes of the ET-synthetic pathway. Enhanced ET levels led to reduced *Arabidopsis* resistance to GPA. Furthermore, dominant suppression of *MYB102* inhibited aphid-induced increase of ET levels in *Arabidopsis*. In agreement with a negative regulatory role for ET in aphid defense responses, the *MYB102-*overexpressing lines were more susceptible to GPA than wild-type (WT) plants. Overexpression of *MYB102* in *Arabidopsis* obviously repressed aphid-induced callose deposition. Conversely, overexpression of *MYB102* failed to increase aphid susceptibility in both the ET-insensitive mutants and plants treated with inhibitors of ET signaling pathways, demonstrating that the ET was critical for promoting aphid performance conferred by overexpression of *MYB102*. Collectively, our findings indicate that the *Arabidopsis MYB102* increases host susceptibility to GPA through the ET-dependent signaling pathways.

## 1. Introduction

Aphids are a large group of phloem-feeding insects, which make use of their highly specialized mouthparts to suck nutrients out of plant sieve elements [1]. Constant attacks of the phloem-feeding aphids cause extensive damage to plant tissues, such as stunted growth, chlorotic leaves, defoliation, and loss of plant yield and quality [2]. Aphids are mainly classified as either specialists or generalists based on their host-plant specificity [3]. For example, mustard aphid (*Lipaphis erysimi*) and cabbage aphid (*Brevicoryne brassicae*) are specialists on cruciferous plants [3], and green peach aphid (GPA, *Myzus persicae*) is a generalist that feeds on a wide range of more than 400 plant species belonging to approximately 50 plant families [4]. GPA is also extremely prolific plant virus vector worldwide, transmitting over 100 plant pathogenic viral species that threaten crop quality and safety [5].

In higher plants, a set of sophisticated strategies has been employed to withstand the attacks from a variety of herbivorous insects during long-term coevolution. Plants have developed constitutive and induced defense tactics, such as physical obstacles (e.g., cuticles, cell walls, and trichomes), and chemical defense (e.g., secondary metabolites) to prevent the infestation of phloem sap feeders [6]. During aphid-host plant interactions, aphids can minimize mechanical wounding and avert triggering intracellular and extracellular defenses by the secretion of gelling and watery saliva [7]. Nevertheless, punctuation of sieve elements by aphid stylets and salivary secretions still provoke active defense responses in host plants [8,9]. Plant defense responses to aphids are modulated by both race-specific resistance and plant basal defense. The race-specific resistant process involves gene-for-gene interaction, in which an effector protein derived from the insect can be recognized by its corresponding host resistance (R) protein, and further activates specific defense pathways to hamper pest invasion [10]. Additionally, plants also own an efficient basal defense system, and this process relies on the perception of wounding signals caused by aphid stylets during the penetration for feeding, and/or and transmission of salivary secretion-derived chemical cues into the host, thereby eliciting the generation of signaling molecules that invoke a common stress response [11,12,13].

Although the defensive strategies vary substantially across different plant species, the defense pathways regulated by hormones are somewhat conserved [14,15,16]. Therefore, it is a pivotal target for compromising plant defense by adjusting the hormone level, timing, and composition. Numerous studies have reported that hormones, such as salicylic acid (SA), jasmonic acid (JA), and ethylene (ET), mount plant defense responses to invading herbivores [17,18,19,20]. However, several pathogens and phloem-feeding insects manage these responses and moderate plant defense network effectively. Many researchers have also unraveled key roles of SA and JA in plant defense against aphids [21,22,23,24,25]. SA signaling is activated by aphid infestation, and transgenic *NahG* tomato plants that are defective in SA synthesis and signaling exhibits increased aphid susceptibility [25]. Phloem-feeding insects can trick hosts into activating SA signaling as a means of suppressing activation of JA signaling [8], indicating that SA and JA exist in an antagonistic relationship during some plant-insect interactions. More recently, abscisic acid (ABA) has been found to be involved in the process of plant-aphid interactions, and negative regulates the resistance of *Arabidopsis* to aphid attacks [26].

The production of ET can be triggered by pathogen and herbivores, but the role of ET for plant defense responses to aphids is largely controversial [23,27,28,29]. Elevation of ET levels activates host defense pathways, as well as promotes viral infection efficiency [5,30]. Induction of ET synthesis by virus or aphid infestation occurs in *Arabidopsis* and tomato, leading to increased host susceptibility to the aphids [5,25,29]. The expression of several genes responsible for ET metabolism and signal transduction are highly upregulated in the aphid-infested plants [30,31]. Aphid-induced ET synthesis is controlled by some genes encoding 1-aminocyclopropane-1-carboxylic acid (ACC) synthase (ACS) that catalyze one decisive step of ET synthesis at the transcriptional level [31]. Regulation of ET signaling can also be operated at the level of perception during aphid-plant interactions [32]. Ethylene response 1 (ETR1) is a positive regulator of ET responses, which is important for effective herbivore infestation [25]. Loss of ETR1 function reduces aphid population sizes in *Turnip mosaic virus*-infested *Arabidopsis*, indicating that ET plays a key role in the resistance of plants to aphid infestation [33].

Recent studies have shown that the members of MYB transcription factor family are involved in regulation of diverse physiological processes, such as plant growth and development, leaf senescence and abiotic and biotic stress responses [34,35,36,37,38]. Several MYB transcription factors play important roles in the defense responses of plants to aphids. Lü et al. [36] have reported that induction of *Arabidopsis MYB44* by aphids can activate the ET signaling pathway by regulation of the ethylene-insensitive protein 2 (EIN2) protein, thereby enhancing the resistance of plants to aphid infestation. In wheat, three *MYB* genes, including *TaMYB19*, *TaMYB29*, and *TaMYB44*, co-regulate the phloem-based defense against aphid attacks [37]. In *Chrysanthemum*, overexpression of *CmMYB19* increases aphid tolerance via promoting the biosynthesis of lignin [38]. In this study, we found that the transcription of *Arabidopsis MYB102* was significantly enhanced in response to GPA infestation. Overexpression of *MYB102* promoted the transcription of some ET biosynthetic genes, thereby increasing ET accumulation. *Arabidopsis* plants ectopically induced to express *MYB102* exhibited higher ET content and were more susceptible to GPA compared with wild type (WT) plants. By contrast, chimeric repressors (SRDXs) of *MYB102* displayed reduced ET levels in the aphid-infected plants. Additionally, high-level expression of *MYB102* could not increase aphid susceptibility in plants defective in the ET signaling pathways. It was thus proposed that *MYB102* negatively regulated *Arabidopsis* resistance to GPA at least partially through the ET-dependent pathways.

## 2. Results

### 2.1. Induction of MYB102 Expression in Arabidopsis by Aphid Infestation

**Figure 1:**
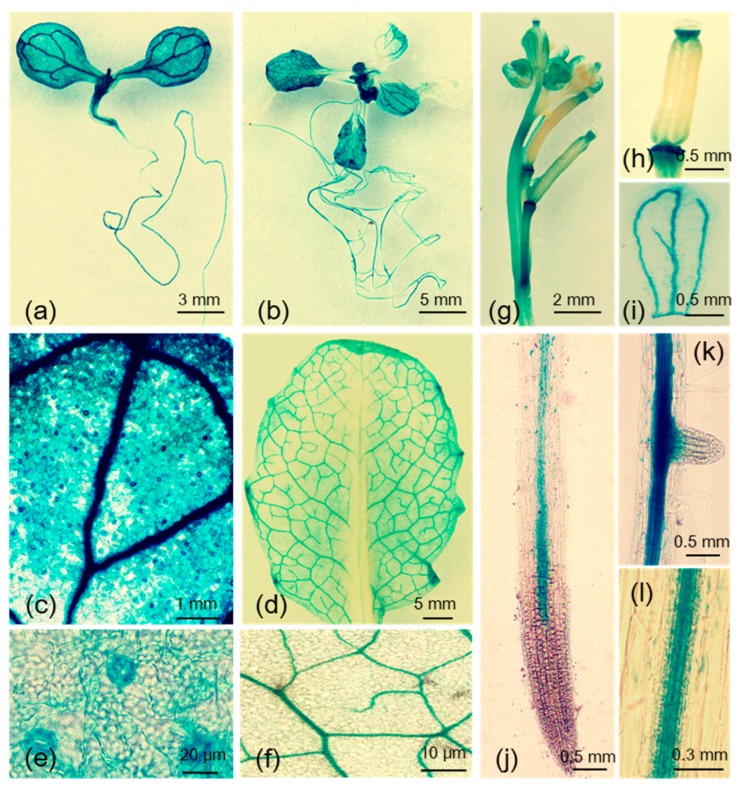
Detection of *MYB102* promoter activity by glucuronidase (GUS) staining. GUS activity driven by *MYB102* promoter in five-day-old seedlings (**a**), 14-day-old seedlings (**b**), vascular tissues in leaves of five- (**c**) and 14-day-old seedlings (**d**), stomata in leaves of 5-day-old seedlings (**e**), leaves of 14-day-old seedlings (**f**), flowers (**g**), siliques (**h**), petals (**i**), primary roots (**j**), lateral roots (**k**), and vascular tissues in roots (**l**).

The *MYB102* gene is developmentally expressed in almost all organs by investigation of public data on the *Arabidopsis* eFP Browser (Figure S1). In this study, histochemical analyses in *pMYB102:GUS* plants revealed that *MYB102* was expressed in almost all organs, such as shoots, roots, flowers, and siliques. In five- and 14-day-old seedlings, the activity of glucuronidase (GUS) was found in shoots and roots (Figure 1a,b), and stronger GUS activity occurred in vascular tissue in leaves (Figure 1c,d). *MYB102* was also highly transcribed in stomata of five-day-old seedlings (Figure 1e), but not in 14-day-old seedlings (Figure 1f). Soon after flowering, GUS staining was found in the top sides of siliques, junction of flower stalks, and veins in petals (Figure 1g–i). Vascular tissues in roots showed strong staining, and no staining was detected in primary and lateral root tips (Figure 1j–l).

**Figure 2:**
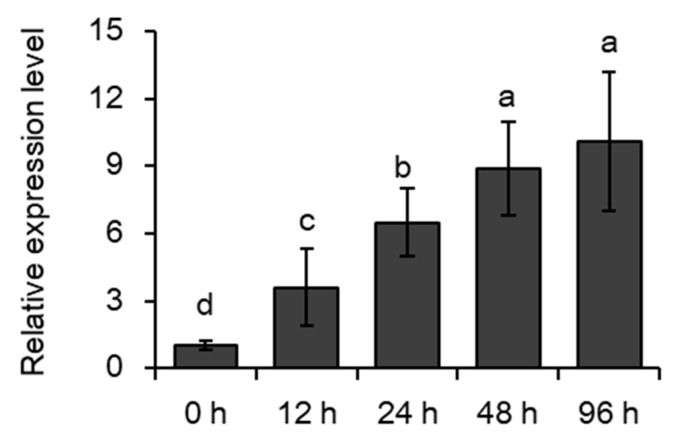
The transcription of *MYB102* in response to aphid infestation. Quantitative Real Time- Polymerase Chain Reaction (qRT-PCR) analysis of *MYB102* expression relative to that of *ACTIN2* in uninfested and aphid-infested leaves of *Arabidopsis* plants carrying *pMYB102:GUS* at the indicated time post-infestation. Values are mean ± SD (standard deviation), *n* = 3. Different letters above bars denote significant difference among different treatments using Tukey’s test at *p* < 0.05.

Moreover, quantitative Real Time-Polymerase Chain Reaction (qRT-PCR) analysis showed that the expression of *MYB102* in the aphid-infested plants differed significantly from those in uninfested plants, and its transcription was gradually increased after aphid infestation (Figure 2).

### 2.2. Overexpression of MYB102 Increases Host Susceptibility to Aphids

**Figure 3:**
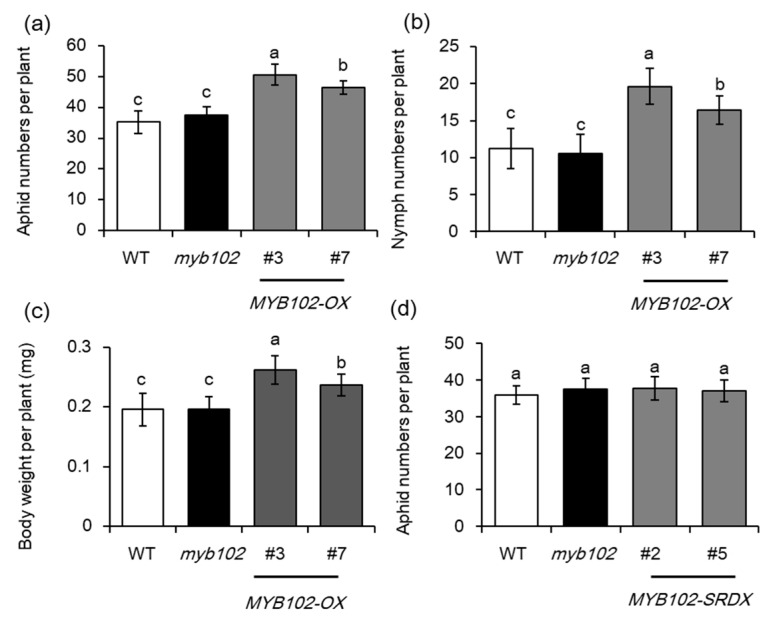
Overexpression of *MYB102* increases *Arabidopsis* susceptibility to aphid infestation. (**a**) Aphid population size (adults plus nymphs) reared on the WT, *myb102*, and *MYB102-OX* lines (*n* = 10) after 48 h of infestation. (**b**) The number of progeny from initially one female per plant was measured after 10 days of nymph infestation (*n* = 10). (**c**) Average body weight of adult aphids on the WT, *myb102*, and two independent *MYB102-OX* and *-SRDX* lines (*n* = 10) after 48 h of infestation (**d**) Aphid population size on the WT, *myb102*, and two independent *MYB102-SRDX* lines (*n* = 10) after 48 h of infestation. Different letters above bars denotes significant difference among different treatments using Tukey test at *p* < 0.05.

To investigate the role of *MYB102* in the responses of *Arabidopsis* plants to GPA, we compared analyses of aphid population growth on the WT, *MYB102-OX* lines, and *myb102* mutants. Plants defend against aphid infestation, which is often reflected by the reduction of offspring production, less feeding, and reduced body weight in no-choice bioassays. In no-choice tests, each plant was infested with 20 adult apterous aphids, and aphid population size (adults plus nymphs) was evaluated 48 h post infestation. *MYB102*-overexpressing (*MYB102-OX*) lines had stronger expression of *MYB102* compared with the WT plants (Figure S2a), and the transgenic lines shared similar phenotypes with the WT plants under normal conditions. These transgenic lines exhibited aphid population sizes that were larger than those on the WT and *myb102* mutants (Figure 3a). No significant difference was observed between both the WT and *myb102* mutants. Overexpression of *MYB102* in *Arabidopsis* induced a great increase of aphid population sizes, due to higher aphid fecundity on the *MYB102*-*OX* lines compared to the WT plants (Figure 3b). These transgenic lines displayed an average of 1.8 nymphs/day, which was significantly higher than 1.2 nymphs/day that were produced on the WT plants. Body weights of aphids feeding on the *MYB102*-*OX* lines were significantly higher than those on the WT plants or *myb102* mutants (Figure 3c). We also generated some chimeric repressor lines (*MYB102-SRDX*) to rule out the possibility of functional redundancy of other transcription factors (TFs). Two independent *MYB102-SRDX* lines displayed higher transcription of *MYB102* than the other lines (Figure S2b). Although the *MYB102-SRDX* lines shared the similarity of aphid population sizes with the WT and *myb102* mutants 48 h post infestation, but the transgenic lines had less body weight than both the WT plants (Figure 3d).

### 2.3. Transcriptomic Analysis of MYB102-Regulated Gene Expression Profiles in Arabidopsis

**Figure 4:**
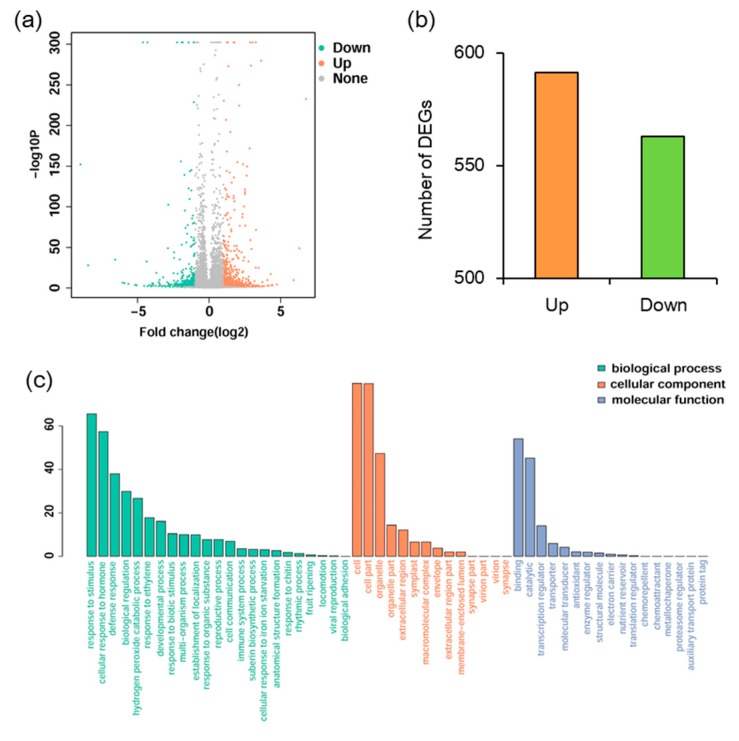
Analyses of differentially expressed genes (DEGs) between the wild-type (WT) and *MYB102-OX3* lines. (**a**) Volcano plot showing up- and down-regulated DEGs. The *x*-axis indicated the value of log2 (OX3/WT), and the *y*-axis indicated the value of −log10 (1-probability). (**b**) Statistics of up- and down-regulated DEGs. The *x*-axis indicated downregulated and upregulated genes, and the *y*-axis indicated the number of DEGs. (**c**) Most enriched Gene Ontology (GO) terms of DEGs were categorized into biological process, cellular component, and molecular function. The *x*-axis indicates GO terms and the *y*-axis indicates the percent of genes in all the DEGs.

**Figure 5:**
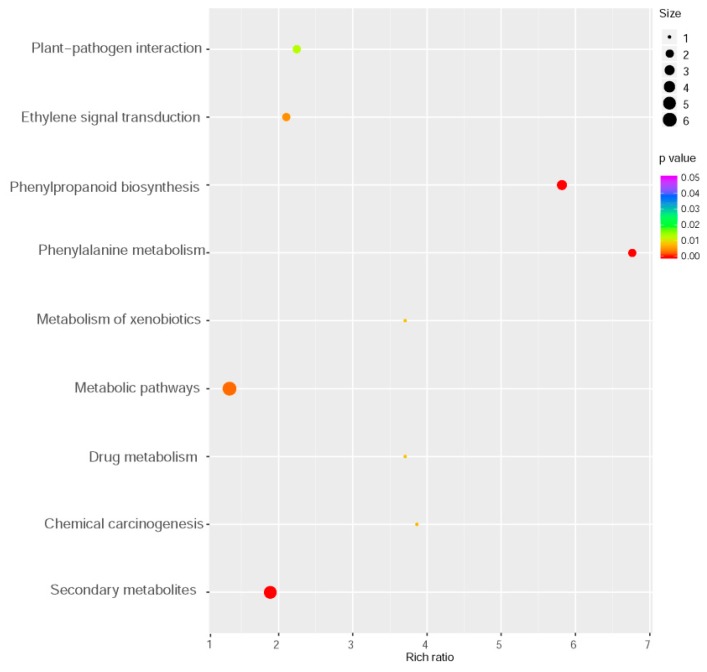
Kyoto Encyclopedia of Genes and Genomes (KEGG) enrichment analysis of differentially expressed genes between the WT and *MYB102-OX3* lines. Size and color of the bubble indicated the amount of DEGs enriched in pathway and enrichment significance, respectively.

To elucidate the mechanism underlying *MYB102* negatively regulated defense responses in *Arabidopsis*, whole genome transcriptional profiles were surveyed by RNA-Sequencing (RNA-Seq). We performed a comparative transcriptomic analysis to identify differentially expressed genes (DEGs) between the WT and *MYB102-OX3* lines. A total of 1154 DEGs (minimal 1.5 log 2-fold change and *p* value < 0.05) were available in Table S1, showing 591 and 563 up- and down-regulated genes, respectively (Figure 4a,b). To examine the functional classification of DEGs, Gene Ontology (GO) enrichment analysis was conducted for these DEGs. GO terms of DEGs were clustered into three main GO categories including biological process, cellular component and molecular function (Figure 4c). The results of enriched analysis revealed that 23, 14, and 17 GO terms were enriched in biological process, cellular component, and molecular function, respectively. The GO categories in biological processes revealed that a large proportion of genes were associated with the pathways involving various stress and stimuli. To further explore the biological pathways of DEGs, the pathway enrichment analysis was carried out. The results showed that nine pathways were significantly enriched (Figure 5). It was noteworthy that some of these enriched genes were positively related to plant-pathogen interaction and ET signal transduction.

To validate the reliability of RNA-Seq data, qRT-PCR was used to detect the transcription levels of 10 randomly selected DEGs including putative flavin-dependent monooxygenase (*FMO1*), SAUR-type hormone signal effector (*SAUR20*), cytochrome P450 monooxygenase (*CYP708A1*), myo-inositol oxygenase (*MIOX2*), thalianol synthase (*PEN4*), β-glycosyl hydrolase (*BGLU31*), putative thionin (*THI2.1*), terpene synthase (*TPS20*), VLCFA-elongase-type 3-ketoacyl-CoA synthase (*KCS15*), and putative ocimene synthase (*TPS3*). The results of qRT-PCR analyses showed that the expression profiles of the tested genes were much similar to those observed in the RNA-Seq data (Figure S3).

### 2.4. Overexpression of MYB102 Affects Endogenous Hormone Levels in Arabidopsis

**Figure 6:**
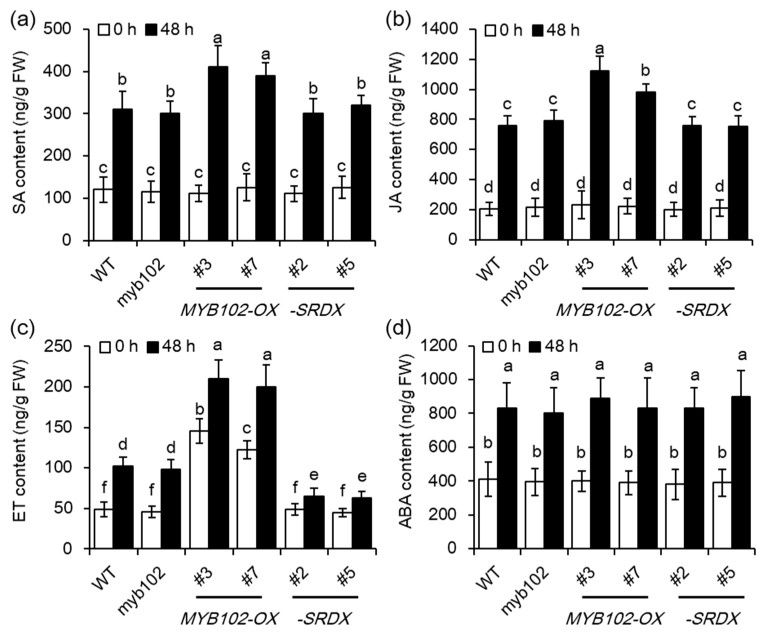
Overexpression of *MYB102* increases ethylene (ET) production in uninfested and aphid-infested *Arabidopsis* plants. The content of salicylic acid (SA) (**a**), jasmonic acid (JA) (**b**), ET (**c**), and abscisic acid (ABA) (**d**) was determined in the WT, *myb102*, *MYB102-OX* and *-SRDX* lines after zero and 48 h of aphid infestation. At least three replicates were conducted for each genotype. Different letters above bars denotes significant difference among different treatments using Tukey’s test at *p* < 0.05.

Phytohormones, including ABA, JA, SA, and ET, play major roles in modulating plant defense responses to aphids [23,24,25,26]. To test whether the compromised plant susceptibility to GPA conferred by up-regulation of *MYB102* was associated with alteration of plant hormones, the content of SA, JA, ET, and ABA were quantified in different genotypes, including WT, *my102*, *MYB102-OX*, and *-SRDX*, respectively. A great increase of ET level was observed in the two *MYB102-OX* lines, while other hormones, including SA, JA, and ABA were not significant different among these genotypes (Figure 6). After 48 h of aphid infestation, the content of hormones was apparently increased in these genotypes, whereas the levels of SA, JA, and ET were higher in the *MYB102-OX* lines than in both the WT and *MYB102-SRDX* lines. Activation of SA- and JA-dependent signaling pathways has been reported to augment the capability of plants to counteract aphid attacks [23,24,25,26]. These results indicated that overexpression of *MYB102* in *Arabidopsis* increased aphid performance, which may be mainly attributed to mediation of ET synthesis or signaling. The data of RNA-Seq also revealed that the transcription of some ET biosynthetic genes including *ACS4*, *ACS7*, *ACS8*, and *ACS11* were significantly upregulated in the *MYB102-OX3* lines (Table S2).

**Figure 7:**
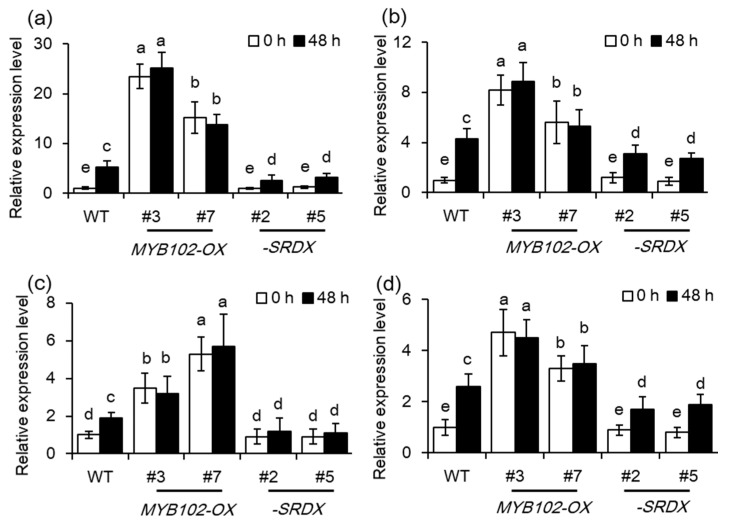
Overexpression of *MYB102* upregulates the expression of ET biosynthetic genes in *Arabidopsis*. Relative expression levels of *ACS4* (**a**), *ACS7* (**b**), *ACS8* (**c**), and *ACS11* (**d**) were examined in the WT, *myb102*, *MYB102-OX* and *-SRDX* lines after 0 and 48 h of aphid infestation. Different letters above bars denotes significant difference among different treatments using Tukey’s test at *p* < 0.05.

Furthermore, the expression levels of these *ACS* genes in the different genotypes were determined using qRT-PCR analyses (Figure 7). The transcription of these *ACS* genes was significantly higher in the *MYB102-OX* lines than the WT plants. Aphid infestation has been found to induce the expression of the *ACS* genes, while dominant repression of *MYB102* notably repressed aphid-induced *ACSs* expression in *Arabidopsis*.

### 2.5. Ethylene Signaling is Crucial for MYB102-Regulated Aphid Susceptibility

**Figure 8:**
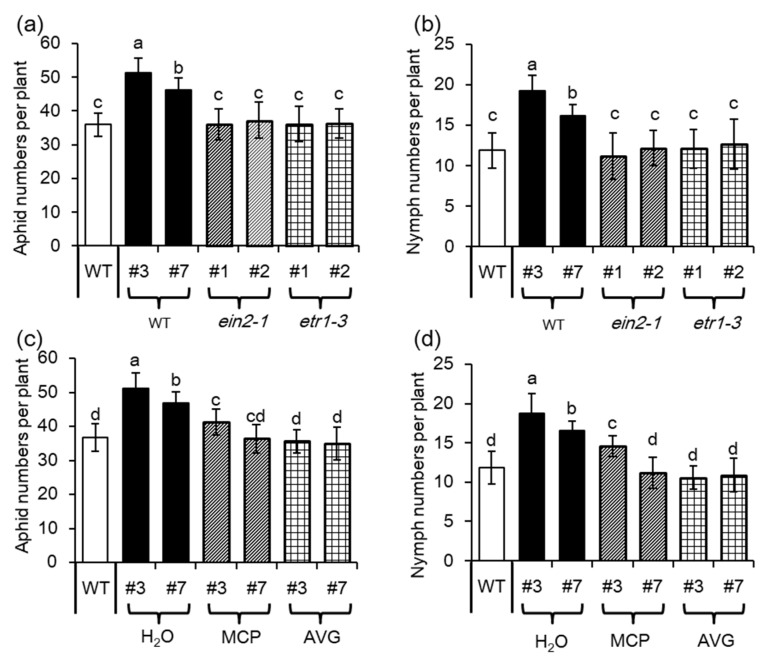
ET signaling is essential for increasing *Arabidopsis* susceptibility to aphids conferred by overexpression of *MYB102*. (**a**) Population size (adults plus nymphs) of adult aphids on WT, *MYB102-OX* lines, *MYB102-OX*/*ein2–1*, and *MYB102-OX*/*etr1–3* (*n* = 10) after 48 h of infestation, and (**b**) the number of progeny nymphs reared on these plants was measured after 10 days of nymph infestation (*n* = 10). (**c**) Population size (adults plus nymphs) of adult aphids on both the WT and *MYB102-OX* lines, MCP- or AVG-treated *MYB102-OX* lines after 48 h of infestation, and (**d**) the number of progeny nymphs was calculated after 10 days of nymph infestation (*n* = 10). Different letters above bars denotes significant difference among different treatments using Tukey’s test at *p* < 0.05.

To investigate the role of ET in *MYB102-*regulated plant defense responses, both the mutants that are insensitive to ET (ethylene insensitive2, *ein2–1*) and ET receptor 1 (*etr1–3*) mutants were used. As shown in Figure 8, overexpression of *MYB102* could not increase aphid population sizes and fecundity in both the *ein2–1* and *etr1–3* mutants, which was much similar to those in the WT plants. Pharmacological approaches were used to compromise the ET signaling pathways. The *MYB102-OX* lines were treated with an inhibitor of ET biosynthesis, aminoethoxyvinyl glycine (AVG), and a chemical agent, 1-methylcyclopropene (MCP), for blocking ET perception, respectively. In this study, 20 adult apterous aphids were released on each plant, allowing them to develop and reproduce for 48 h. Aphid population sizes and fecundity in the *MYB102*-*OX* lines were remarkably decreased when plants were treated with the inhibitor of ET biosynthesis (AVG) or perception (MCP), favoring the results that were observed in the genetic approaches.

**Figure 9:**
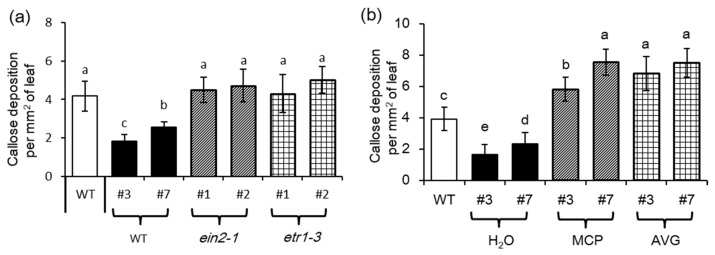
ET signaling is required for inhibiting callose accumulation by overexpression of *MYB102.* (**a**) Callose deposition in leaves of WT, *MYB102-OX* lines, *MYB102-OX*/*ein2–1*, and *MYB102-OX*/*etr1–3* (*n* = 10) after 48 h of aphid infestation. (**b**) Callose deposition in leaves of WT and *MYB102-OX* lines with or without treatments with 1-methylcyclopropene (MCP) or aminoethoxyvinyl glycine (AVG). Different letters above bars denotes significant difference among different treatments using Tukey’s test at *p* < 0.05.

*Turnip mosaic virus* (TuMV)-induced increase of ET levels is vital for inhibiting callose deposition caused by aphid attacks [33]. Consistently, the *MYB102*-*OX* lines with higher ET content displayed less callose accumulation than the WT plants. However, overexpression of *MYB102* strikingly stimulated callose deposition in the two mutants (*ein2–1* and *etr1–3*) defective in the ET signaling pathways (Figure 9a). Aphid-induced accumulation of callose in the *MYB102*-*OX* lines was also quantified when plants were treated with the chemical inhibitors AVG and MCP, as described above. Aphid-induced callose production was observably decreased in the *MYB102*-*OX* lines (Figure 9b). By contrast, the ET-signaling inhibitors resulted in an increase of callose deposition in the aphid-infected plants. Accordantly, overexpression of *MYB102* promoted more accumulation of callose in the ET signaling-defective mutants compared to that in the WT plants, indicating that the increased ET levels conferred by overexpression of *MYB102* may contribute to inhibition of callose formation during plant-aphid interactions.

## 3. Discussion

Aphid infestation can stimulate ET synthesis in different plant-aphid interactions [27,28,29]. An increase of ET levels has been shown to promote aphid susceptibility in several plant species, such as tomato [29] and *Arabidopsis* [33]. Similarly, it was observed in this study that the ET production in *Arabidopsis* was greatly induced by aphid feeding. Compared with WT plants, overexpression of *MYB102* compromised host resistance to aphid infestation, displaying better performance of GPA on these transgenic lines. Overexpression of *MYB102* significantly promoted the ET biosynthesis in uninfested plants. However, dominant repression of *MYB102* did not affect the content of ET in uninfested plants. After aphid infestation, the *MYB102-SRDX* lines had lower ET accumulation than the WT plants. Our findings clearly demonstrated that induction of ET production by *MYB102-*overexpression attenuated host defense mechanisms with less callose deposition, while disruption of ET synthesis or signaling reinstated host effective defense against the invading aphids. Hence, these data confirmed that induction of ET production was beneficial to aphid colonization, and the aphids may hijack host defense response via activation of *MYB102* expression and, thus, intensify ET synthesis or signaling, thereby controlling host defense responses. These findings also deepen our understanding of the mechanisms that modulation of host ET production by the aphids may decrease plant defense response towards aphids. 

ET functions in the regulation of various physiological processes such as seed germination and leaf senescence as well as abiotic and biotic stress responses [39,40,41,42]. During abiotic stress, ET signaling is required for plant adaption to salt and drought stress [43,44], nevertheless overproduction of cellular ET reduces the tolerance of plants to the stress [45,46,47]. A generalized role of ET has not been established during biotic stress. ET plays key roles in assisting plants to prevent the infection of necrotrophic pathogens [48], but ET also functions as negative signaling molecules in other case of plant-pathogen interaction [30]. Increasing evidence has indicated that the functions of ET in regulation of plant defense response vary significantly with specific host species, and are also mediated by the crosstalk between various signals [49]. *Rice dwarf virus* (RDV)-triggered ET production increases host susceptibility to RDV by enhancing the S-adenosyl-L-methionine synthetase activity [30]. *Arabidopsis ein2* and *etr1* mutants that are defective in ET signaling pathways exhibit stronger resistance to *Cauliflower mosaic virus* (CaMV) [50] and *Tobacco mosaic virus* (TMV) [30] than WT plants. Another recent study found that induction of ET by *Turnip mosaic virus* (TuMV) enhanced virus infection efficiency, but was greatly impaired in the *ein2* and *etr1* mutants, indicating that ET signaling was responsible for weakening host defense response by virus [33]. Aphid-induced increases of ET levels also occur in different plant species [25,29], although ET signaling contributes to increased host susceptibility and resistance to aphids [23,27,28,29]. Although the role of ET in *Arabidopsis*-aphid interactions has been established [33], the underlying mechanism of aphid-regulated ET biosynthesis is rarely understood, with only little information on the involvement of ET signaling in plant-aphid interactions.

To take deep insight into the mechanism, we identified a R2R3-MYB transcription factor gene *MYB102* from *Arabidopsis thaliana,* probably involving regulation of plant defense response. The *Arabidopsis MYB102* has previously been reported to respond rapidly to various stresses such as wounding, salinity, and dehydration, as well as hormones including ABA and JA [51]. Moreover, *MYB102* has been shown to positively regulate the defense response of *Arabidopsis* to the insect herbivore *Pieris rapae* [52]. In this study, we found that *MYB102* was expressed preferentially in vascular tissues of leaves and roots. Tissue-specific expression profiles of *MYB102* was much similar to those of several genes, such as actin-depolymerizing factor 3 (*ADF3*), indole glucosinolates (IG) synthetic genes *CYP79B2* and *CYP79B3* responsible for confronting aphid infestation [53,54]. The transcription of *MYB102* was remarkably increased following aphid infestation, and overexpression of *MYB102* compromised *Arabidopsis* defense against the GPA. Conversely, knockout of *MYB102* in *Arabidopsis* reduces the tolerance to caterpillar feeding [52]. Lei et al. [55] have reported that the *Arabidopsis BIK1* negatively regulates the tolerance to aphids, but not to chewing insects. *Aphis craccivora* (sucking pest) or *Helicoverpa armigera* (chewing insect) infestation leads to differential profiles of flavonoids in *Arachis* plants, indicating that plants respond to the sucking pest and chewing insect with different modes of action [56]. In addition, potato plants challenged with a piercing-sucking insect *Myzus persicae* Sultzer (sucking pest) or *Leptinotarsa decemlineata* Say (chewing insect) display differential changes in defense metabolites, such as volatile compound release and oxylipin synthesis [57]. Hence, *MYB102* may differentially regulate defense strategies to withstand the attacks by different insect predators.

At least four phytohormones, SA, JA, ABA, and ET, have been demonstrated to orchestrate plant response to aphids [23,24,25,26]. SA is able to activate plant defense responses against a broad-spectrum of pathogens [20], but it seems to be dispensable for limiting aphid infestation on *Arabidopsis* [8]. JA signaling has major functions in resisting necrotrophic fungal pathogens, virus and herbivores [13,14,15]. In addition, elevation of cellular ABA and ET can reduce the resistance of *Arabidopsis* plants to GPA [26,33]. In this study, overexpression of *MYB102* significantly increased ET levels in host plants, while the content of other hormones was not observably changed. However, the levels of SA, JA, and ET were noticeably higher in the *MYB102-OX* lines than those in both the WT and *MYB102-SRDX* lines upon exposure to aphid infestation. It was thus supposed that the compromised GPA susceptibility was not likely due to the increases of SA and JA levels, while was primarily as consequence of elevated ET production. It has recently been indicated that viral infection greatly suppressed the aphid-induced callose accumulation in *Arabidopsis* plants [33]. ET has been demonstrated to be required for inhibiting the accumulation of callose triggered by virus. Casteel et al. [33] have reported that disruption of ET biosynthesis or signal transduction recovered aphid-induced callose deposition in the virus-infected *Arabidopsis*. Reduction of callose production has been shown to weaken plant defenses against aphids, which contributes to more aphid fecund on the plants [33]. Thus, we speculated that overexpression of *MYB102* in *Arabidopsis* considerably increased ET accumulation and further inhibited aphid-induced callose deposition, which resulted in increased host susceptibility to aphids. Consistently, overexpression of *MYB102* could not increase aphid susceptibility in the *ein2* and *etr1* mutants with more callose accumulation compared with that in the WT plants. Similar results were also shown in the plants treated with chemical inhibitors of ET biosynthesis (AVG) or perception (MCP). In addition, that the expression of some ET biosynthetic genes including *ACS4*, *ACS7*, *ACS8* and *ACS11* were significantly activated in the *MYB102*-*OX* lines. However, dominant repression of *MYB102* significantly inhibited the expression of these ET biosynthetic genes and reduced ET content in the aphid-infested *Arabidopsis*. Thus, the *Arabidopsis MYB102* may positively regulate the expression of these *ACS* genes and further increase ET accumulation, thereby reducing the resistance of plants to the aphids. *ACS4* has previously been found to be expressed in vascular tissues, and is significantly up-regulated by heat stress, auxin, and wounding signals [58]. *ACS7* is primarily expressed in protoxylem of vascular tissues [58], and its function loss reduces ethylene emission in *Arabidopsis* with higher tolerance to various abiotic stresses, such as salt, osmotic, and heat stresses [59]. Dong et al. [60] have shown that ABA negatively regulates ET synthesis in *Arabidopsis* via transcriptional repression of *ACS4* and *ACS8*. Additionally, the expression patterns of *ACS11* are similar to that of *ACS8*, yet its transcriptional intensity is relatively higher [58]. Therefore, it may be inferred that aphid infestation triggered ET production, at least partially, by activation of *MYB102*-regulated *ACSs* expression.

In summary, the elevated ET levels promoted aphid performance on *Arabidopsis* plants by mediating plant defense response. Overexpression of *MYB102* enhanced the ET-dependent susceptibility of host plants to GPA. The defects in ET signaling pathway failed to suppression of plant defense against the aphids conferred by overexpression of *MYB102*, thus, lowering ET production or disrupting ET signaling pathways increased host resistance to aphid infestation.

## 4. Materials and Methods

### 4.1. Plant and Aphid Materials

Seeds of *Arabidopsis thaliana myb102* (SK35587), *ein2–1* (CS3071) and *ert1–3* (CS3070) mutants [33] were used in this study. The full-length coding region of *MYB102* was obtained by Polymerase Chain Reaction (PCR) amplification using a pair of gene-specific primers, and was inserted into the binary vector pCambia1301 to generate recombinant vectors for *MYB102*-overexpressing lines. To construct dominate repressors of *MYB102*, the coding region of *MYB102* without terminator codon sequence was fused to specific sequences encoding a putative SRDX domain (LDLDLEL) [61], and was then inserted into the pCambia1301. The promoter sequence of *MYB102* was inserted into the binary vector pCambia1304 to generate the vector *pMYB102:GUS*. Lastly, these constructs were introduced into *Arabidopsis thaliana* by the *Agrobacterium*-mediated floral dip transformation. *MYB102-OX* and *-SRDX* transgenic lines were verified by PCR, and T3 homozygous lines were selected for next experiments. All plants were grown in a growth chamber with a 16-h-light (100 μmol·m^−2^·s^–1^)/8-h-dark cycle at 25 °C and 65% humidity. A GPA colony was collected from the field-grown radish and maintained on *Arabidopsis* plants with a 16-h-light/8-h-dark photoperiod at 25 °C (1000 lux) for all aphid-infested assays. Primers used in the above experiments are listed in Table S3.

### 4.2. Aphid Bioassays and Treatments with Ethylene Inhibitors

No-choice tests were used to assay the growth of GPA reared on both the WT and mutants as described by Mondal et al. [53]. Twenty adult asexually-apterous aphids were placed on leaves of four-week old *Arabidopsis* plants for monitoring aphid populations. After 48 h of aphid infestation, the number of aphids settled on plants was counted. To assess aphid fecundity, one adult aphid was placed on rosette leaves of *Arabidopsis*. After 24 h of aphid infestation, mere one nymph was allowed to settle on leaves and develop for 10 days, and then the number of progeny was recorded. To investigate the role of ET in *MYB102*-regulated defense responses, plants were treated with chemical inhibitors of MCP and AVG to inhibit ET signaling and synthesis, respectively, as described recently by Casteel et al. [33].

### 4.3. Measurement of Hormone Content

The content of SA, JA, and ABA in plants was determined using the isotope-labeled internal standard method as described recently by Lei et al. [55]. Briefly, four-week-old *Arabidopsis* plants were infested with adult aphids (30 per plant) for 48 h. About 500 mg of the uninfested or aphid-infested shoots were ground with liquid nitrogen into powder before addition of 5 mL extraction buffer (HCl:water:isopropanol (2:1:0.005, *v*/*v/v*)). The mixture was vibrated for 30 min at 4 °C, and was then added into 10 mL dichloromethane vibrated for 30 min at 4 °C. Subsequently, the extract was centrifuged at 12,000 rpm for 10 min (4 °C). The denser organic phase was dried with nitrogen, and dissolved in 0.5 mL methanol:0.1% methanoic acid (60:40, *v*/*v*). Then, the extracted solution was analyzed using liquid chromatography-tandem (LC)-mass spectrometry (MS). D4-SA, D5-JA, and D6-ABA (CDN Isotopes, Point-Claire, QC, Canada) were used as internal standards. Additionally, to measure the content of ET, four-week-old *Arabidopsis* plants were exposed to aphid (30 per plant) infestation. The uninfested or aphid-infested shoots were separated and kept in 15-mL syringes. One milliliter of headspace gas was injected into a gas chromatograph (GS), and six independent samples were used for each treatment, as described by Lei et al. [55].

### 4.4. RNA-Sequencing Analyses

Seeds of WT and *MYB102-OX3* lines were surfaced-sterilized and germinated on Murashige and Skoog (MS) agar plates. Then, seven-day-old seedlings were transferred to 1/2 MS liquid medium and grown for three weeks in a growth chamber with a 16-h-light (100 μmol·m^−2^·s^–1^)/8-h-dark cycle at 21 °C. These seedlings were used to extract total RNA for RNA-Seq analyses. The quality and quantity of RNA samples was examined using the Agilent 2100 Bioanalyzer (Agilent, Santa Clara, CA, USA). Five-hundred nanograms of total RNA from each sample was used to generate two cDNA libraries and perform RNA-Seq at Mega Genomics (Beijing, China), and three independent replicates were sequenced. The cDNA libraries with average length of 250-bp cDNA fragments were generated using the Illumina Technology (Illumina, San Diego, CA, USA). The RNA-Seq raw data was filtered to eliminate some adaptor sequences and low-quality mRNA reads, and were deposited into the National Center for Biotechnology Information Sequence Read Archive database (no. SRP141296). DEGs between both WT and *MYB102-OX3* lines were screened with a threshold value of (FDR < 0.05) and |log_2_Fold Change (FC)| ≥ 1.5. Furthermore, the GO functional and KEGG enrichment analyses were conducted by AgriGO6 and KOBAS, respectively.

### 4.5. Quantitative Real Time Reverse Transcription Polimerase Analyses

Four-week-old *Arabidopsis* plants were infested with aphids for indicated times. Then, the aphids were removed from infested leaves, and the uninfested and aphid-infested leaves were immediately harvested to extract total RNA using Trizol reagent (Sangon, Shanghai, China). These plants were grown in a growth chamber with a 16-h light (100 μmol·m^−2^·s^–1^)/8-h dark cycle at 25 °C and 65% humidity. Moreover, residual DNA in total RNA was eliminated by the DNase (Takara, Dalian, China). Approximate 500 ng of total RNA was reversely transcribed into single-strand cDNA using the PrimeScript™ RT Master Mix (Takara, Dalian, China) following the manufacturer’s instructions. Then, cDNA samples were used as the templates of qRT-PCR to examine transcript abundance of target genes including *MYB102, FMO1, SAUR20, CYP708A1, MIOX2, PEN4, BGLU31, THI2.1, TPS20, KCS15*, and *TPS3.* qRT-PCR reaction was carried out using the TBGreen™Premix ExTaq™II (Takara, Dalian, China) in an Applied Biosystems (ABI) 7500 PCR machine (Applied Biosystems, Carlsbad, CA, USA) according to the methods reported by Zhou et al. [62]. To normalize gene expression levels, the *ACTIN2* gene was selected as an internal control. Primers used in above experiments were listed in Table S3.

### 4.6. Glucuronidase and Callose Staining

*Arabidopsis* plants carrying *pMYB102:GUS* were stained by histochemical analyses according to the methods described by Zhou et al. [62]. Plant samples were incubated with the staining solution containing 0.1 M sodium phosphate buffer (pH 7.2), 0.1% (*v*/*v*) Triton X-100, 2 mM potassium ferrocyanide and potassium ferricyanide, 2 mM 5-bromo-4-chloro-3-indolylglucuronide (X-Gluc) for 16 h at 37 °C. Then, these samples were bleached with 70% (*v*/*v*) ethanol in triplicate, and were photographed under a Nikon Eclipse 80i microscope (Tokyo, Japan).

For callose staining, 20 adult asexually-apterous aphids were placed on rosette leaves of *Arabidopsis* plants, and leaf tissues were harvested after 24 h of infestation. Callose deposition was observed according to the methods described by Casteel et al. [33]. Leaf samples were initially decolored with 95% (*v*/*v*) ethanol, and were then immersed in the staining solution containing 0.1 M K_2_PO_4_ (pH 9.5) and 0.05% (*v*/*v*) aniline blue for one hour. Lastly, the staining leaves were observed by a Leica confocal microscope (Leica, Wetzlar, Germany), and the staining spots were recorded.

### 4.7. Statistical Analysis

SPSS 16.0 software (SPSS IBM, New York, NY, USA) was used for analyzing all data. The no-choice tests of aphid performance among different genotypes were examined using one-way ANOVA. Tukey’s multiple range test analysis was used for pairwise comparisons of the difference between treatments at *p* < 0.05. All assays of aphid population size, aphid fecundity, and callose deposition were repeated at least three times and included 10 experimental units per treatment. qRT-PCR experiments were performed in triplicate, each with three biological repeats.

## Supplementary Materials

The following are available online at http://www.mdpi.com/2218-273X/8/2/39/s1; Figure S1. Relative expression of *MYB102* in *Arabidopsis thaliana* during the whole life cycle; Figure S2. qRT-PCR analyses of *MYB102* expression in different *MYB102-OX* lines (**a**) and *MYB102-SRDX* lines (**b**); Figure S3. qRT-PCR validation of differentially-expressed genes (DEGs) corresponding to the data of RNA-Seq. The expression levels of 10 randomly selected genes from the DEGs were examined; Table S1. Upregulated and downregulated DEGs between the WT and *MYB102-OX3* lines grown on 1/2 MS medium for four weeks; Table S2. Upregulation of ET biosynthetic genes in the *MYB102-OX3* lines; Table S3. All the primers that were used in this study.
