# Supplementary figures and images for: Arabidopsis Transcription Factor MYB102 Increases Plant Susceptibility to Aphids by Substantial Activation of Ethylene Biosynthesis
Source: Biomolecules. 2018 Jun 7;8(2):39. doi: 10.3390/biom8020039 (PMC6023100; doi:10.3390/biom8020039)

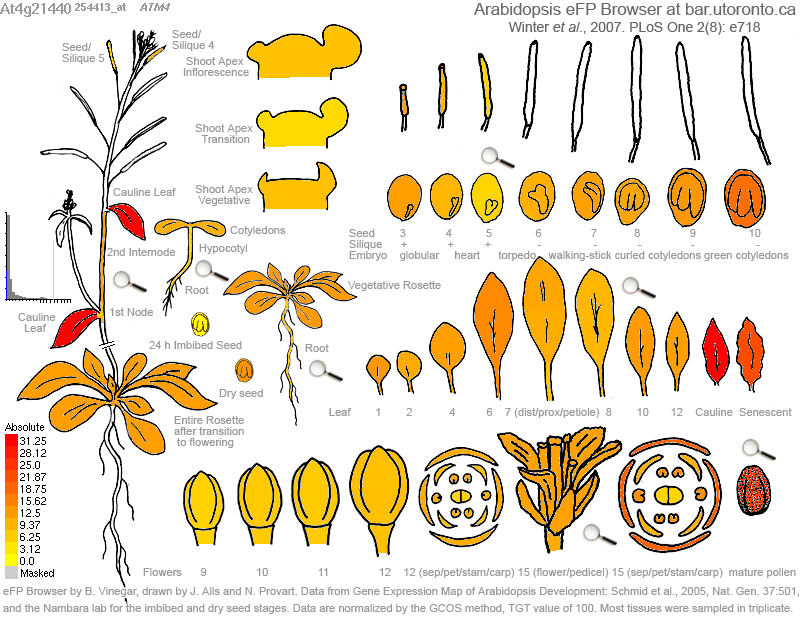

Supplement: Supplementary file 1 [file biomolecules-08-00039-s001.zip › Figure S1.png]

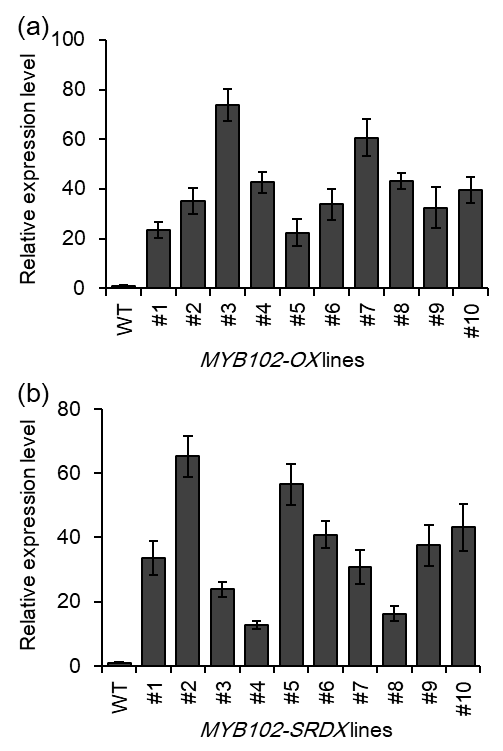

Supplement: Supplementary file 1 [file biomolecules-08-00039-s001.zip › Figure S2.tif]

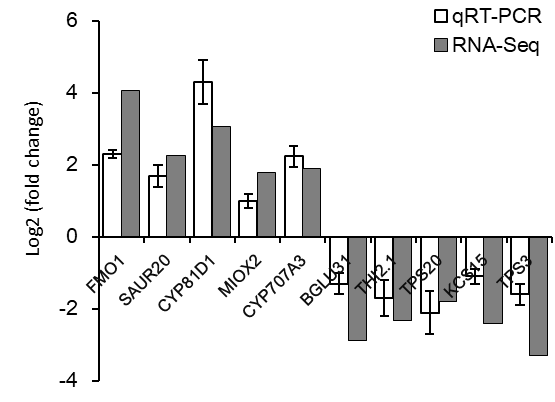

Supplement: Supplementary file 1 [file biomolecules-08-00039-s001.zip › Figure S3.tif]
